# Study of the Effect of Grafting Method on Surface Polarity of Tempo-Oxidized Nanocellulose Using Polycaprolactone as the Modifying Compound: Esterification *versus* Click-Chemistry

**DOI:** 10.3390/nano3040638

**Published:** 2013-12-12

**Authors:** Abdelhaq Benkaddour, Khalil Jradi, Sylvain Robert, Claude Daneault

**Affiliations:** 1 Lignocellulosic Materials Research Center, University of Quebec at Trois-Rivières, 3351 Boulevard des Forges, Trois-Rivières, QC G9A 5H7, Canada; E-Mails: khalil.jradi@uqtr.ca (K.J.); sylvain.robert@uqtr.ca (S.R.); 2 Canada Research Chair in Value-Added Papers, 3351 Des Forges, Trois-Rivières, Québec G9A 5H7, Canada; E-Mail: claude.daneault@uqtr.ca

**Keywords:** nanocellulose, grafting, polycaprolactone, esterification, click-chemistry

## Abstract

Esterification and click-chemistry were evaluated as surface modification treatments for TEMPO-oxidized nanocelluloses (TONC) using Polycaprolactone-diol (PCL) as modifying compound in order to improve the dispersion of nanofibers in organic media. These two grafting strategies were analyzed and compared. The first consists of grafting directly the PCL onto TONC, and was carried out by esterification between hydroxyl groups of PCL and carboxyl groups of TONC. The second strategy known as click-chemistry is based on the 1,3-dipolar cycloaddition reaction between azides and alkyne terminated moieties to form the triazole ring between PCL and TONC. The grafted samples were characterized by transmission electron microscopy (TEM), Fourier transform infrared spectroscopy (FTIR), X-ray photoelectron spectroscopy (XPS), and Thermogravimetry analysis (TGA). Further, the effects of the two treatments on the surface hydrophobization of TONC were investigated by contact angle measurements. The results show that both methods confirm the success of such a modification and the click reaction was significantly more effective than esterification.

## 1. Introduction

Nanocelluloses are cellulosic elements having unit sizes of less than 100 nm in at least one dimension. Several recent and current studies in the literature reveal that nanoscale celluloses are being studied intensively for potential utilization in a range of applications such as biomedicine [1,2], biomaterials engineering [3,4,5,6,7], membranes [8,9], and polymer nanocomposites [10,11,12,13]. According to Schadler *et al.* [14], a polymer nanocomposite is a matrix composite in which the fillers are less than 100 nm in at least one dimension. In the case of nanocelluloses as fillers or reinforcements, the resulting nanocomposites are referred to as cellulose nanocomposites or nanocellulose composites. In depth studies have been conducted on the utilization of bacterial cellulose [15,16], nanocrystalline celluloses from acid hydrolysis [17,18] and micro or nanofibrillated celluloses from mechanical disintegration [19], as reinforcements in polymer nanocomposites. The efficiency of nanofiller dispersion in the matrix is extensively known to critically affect nanocomposite physical and mechanical properties. However, there are some limitations concerning the use of this cellulosic nanomaterial. In fact, the strong hydrophilic behaviour of cellulose has a tendency to form hydrogen bonds between adjacent fibrils (self-aggregation) and reduces the interaction with the hydrophobic molecules and non-aqueous media [20]. In the case of cellulose nanocrystals (CNCs) from acid hydrolysis, they do naturally occur as bundles of rod-like crystallites although intense mechanical agitation is needed [21]. In the case of microfibrillated celluloses (MFCs), they continue to exist as interconnected nanofibrillar structures even after intense attrition from multiple passes through high intensity homogenizers [22]. These limitations stem primarily from the high affinity of nanocelluloses for water and their inability to disperse readily in organic solvents. To overcome this problem, a massive variety of chemical modification techniques including coupling hydrophobic small molecules [23,24,25], grafting polymers and oligomers [26,27], and adsorbing hydrophobic compounds [28,29] to the surface of nanocelluloses were employed.

Recently, according to Saito *et al.* [30,31], it was reported that 2,2,6,6-tetramethylpiperidine-1-oxyl(TEMPO)-mediated selective oxidation of primary alcohols of cellulose fibers is an alternate promising route to obtain individualized microfibrils. In contrast to conventional CNCs and MFCs, TEMPO-oxidized nanocelluloses (TONC) represent a different form of highly individualized and multi-functionalized nanocelluloses, which was attributed to aqueous medium repulsion due to their high surface charge densities. Given that TONC possess multiple advantages, such as, multifunctionality of surface nanofibers and stability of structural integrity of cellulose (the co-existence of crystalline and amorphous domains), we perceived potential advantages in the utilization of TONC as nanocomposite reinforcements.

In the present work, we used at first, a high-intensity ultrasound (170 kHz) in combination with a TEMPO system to oxidize the primary hydroxyl groups on the cellulose to carboxylate groups in order to produce TONC. Then, we converted the carboxyl groups on TONC surfaces into hydrophobic groups for the promotion of TONC dispersion in organic media. So, TONC surfaces were derivatized with polycaprolactone (PCL) as a model hydrophobic compound. Two main pathways to attach PCL to TONC surfaces were evaluated: (1) covalent coupling through esterification reactions; and (2) copper (I)-catalyzed Huisgen 1,3-dipolar cycloaddition (click-chemistry). Unfortunately, the grafting of PCL by esterification did not exceed 5% *w*/*w* and the grafting was not sufficient to yield a hydrophobic composite. This modest grafting yield was attributed to the large molecular weight of PCL inducing an important steric hindrance and thus negatively affecting the grafting density.

To overcome this limitation, we have used a new grafting way developed by Barner-Kowollik *et al.* [32], called click-chemistry. This strategy consists in moving away the PCL macromolecules from the nanofiber’s surface intercalating a spacer molecule between TONC and the PCL chains. Thus, an alkyne-terminated spacer molecule, 10-Undecyn-1-ol was grafted onto TONC by esterification reaction, followed by its reaction with PCL via click-chemistry.

The target of this work was to graft high quantities of PCL chains onto TONC. Thus, two specific objectives were set to achieve the main goal. The first one was to evaluate the efficiency of (1) and (2) as treatment methods for the coupling of PCL to TONC surfaces and the second was to evaluate the effects of (1) and (2) on the surface polarity of TONC.

**Figure 1:**
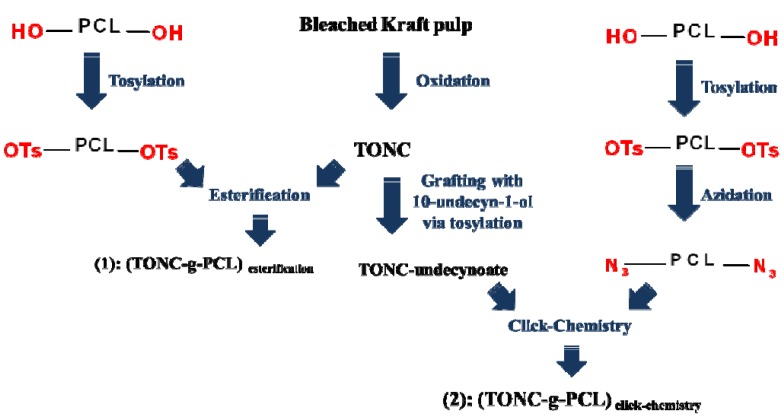
Strategy of TEMPO-oxidized nanocelluloses (TONC) modification adopted in this study.

The strategy of TONC modification adopted in this study is illustrated in Figure 1.

## 2. Results and Discussion

Conductometric titration was used to obtain the degree of oxidation of TONC before (*DO*) and after (*DO*1) coupling with 10-Undecyn-1-ol.

The coupling yield was then calculated using the following Equation:

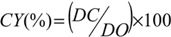
(1)
where *DC* = *DO* − *DO*1.

The degree of oxidation (*DO*) was equal to 0.25, and is given by the following Equation:

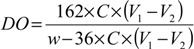
(2)
where *C* is the NaOH concentration (mol/L), *V*_1_ and *V*_2_ are the volume of NaOH (in Litre) required to neutralize the excess protons of hydrochloric acid and those attached to the carboxylic groups, respectively, *w* is the weight of the oven-dried sample (g), 36 is the difference between the molecular weight of the anhydroglucose unit and that of the sodium salt of a glucuronic acid and 162 is the molecular weight of one anhydroglucose unit.

The *DO*1 and *CY* (%) for TONC-Undecynoate were equal to 0.17 and 32, respectively. For both methods, the reactions yields were found to be around 5% and 20% for direct esterification and click reaction, respectively. These values were determined by gravimetry using the following Equation:

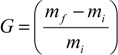
(3)
where *G* is the weight gain, mf and mi are the weights of TONC after and before the grafting respectively.

### 2.1. Fourier Transform Infrared Spectroscopy (FTIR) Experiments

**Figure 2:**
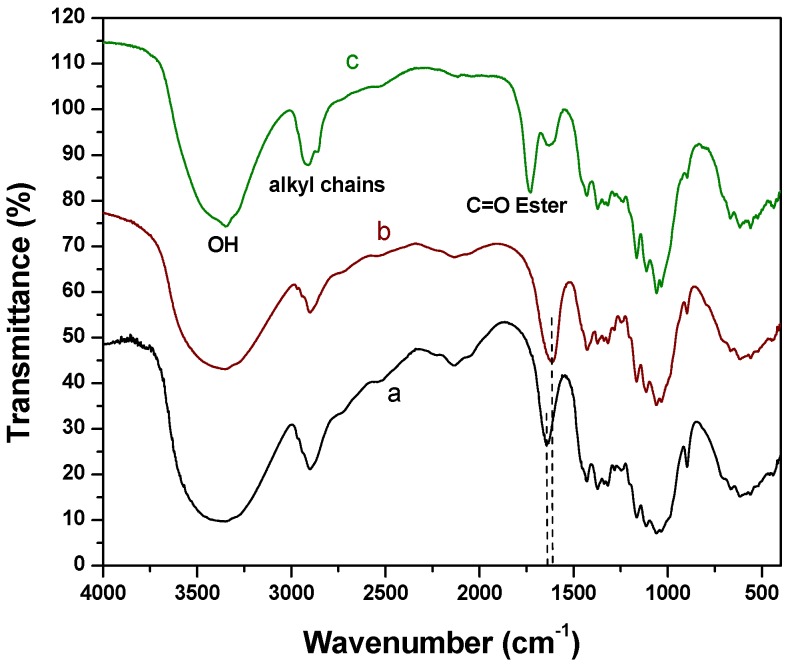
Fourier transform infrared spectroscopy (FTIR) spectra of Kraft pulp (**a**); Tempo-oxidized nanocelluloses (TONC) alone (**b**) and TONC-Undecynoate (**c**).

FTIR spectroscopy was used to compare TONC fibers before and after grafting. Figure 2 shows the coupling of TONC by 10-Undecyn-1-ol. The deconvolution of the peak at 1610–1640 cm^−1^ (spectrum b) shows two others peak. The first at 1610 cm^−1^, characteristic of the C=O band of TONC, which confirms the selective oxidation of the primary alcohol of cellulose. The second band at 1638 cm^−1^ represents the OH bending of adsorbed H_2_O. The FTIR spectrum of dry TONC-Undecynoate (spectrum c) shows the appearance of the band at 1731 cm^−1^, characteristic of ester O–C=O moiety. No decrease was noticed in the signal corresponding to the OH functions appearing around 3365 cm^−1^, whereas, a significant decrease in the signal at 1610 cm^−1^ was observed. This decrease was due to the formation of an ester bond between the carboxylate group of TONC and the alcohol group of 10-Undecyn-1-ol as a result of the formation of TONC-Undecynoate. Moreover, the C≡CH signal expected to appear at 3300 cm^−1^, was found to be overlapped by the peak corresponding to hydroxyl group. In addition, this coupling was also confirmed by the increase of the signal at 2900 cm^−1^ corresponding to the alkyl chains of the spacer molecule (10-Undecyn-1-ol) which contain nine methylene carbons.

**Figure 3:**
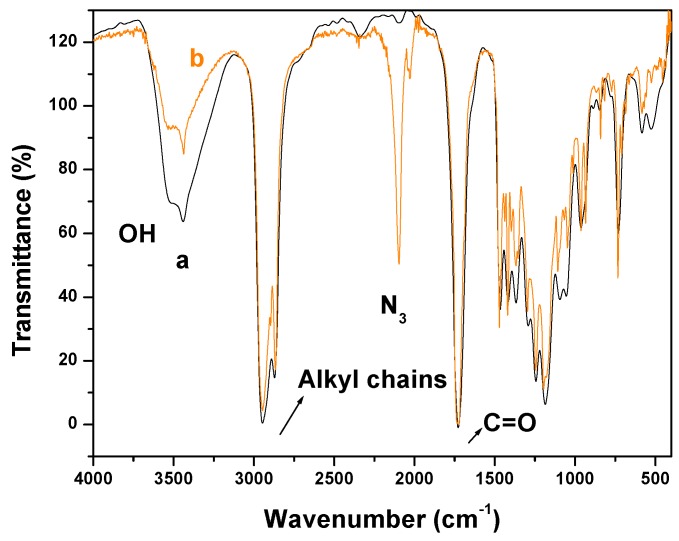
FTIR spectra of PCL (**a**) and azido-polycaprolactone (PCL-N3) (**b**).

Figure 3 shows the FTIR spectra of PCL and PCL-N3. The spectrum of PCL (spectrum a) shows a peak around 3500 cm^−^^1^ and a very sharp signal at 1750 cm^−^^1^, corresponding to hydroxyl and ester groups, respectively. When tosylated polycaprolactone reacted with NaN_3_, the FTIR spectrum of PCL-N3 (spectrum b) showed a considerable decrease in the intensity of the hydroxyl groups at 3500 cm^−^^1^ and the appearance of a new intense band at 2096 cm^−^^1^ typical of the azide groups, which confirms clearly that more and more azide molecules are covalently coupled to the surface of PCL chains.

**Figure 4:**
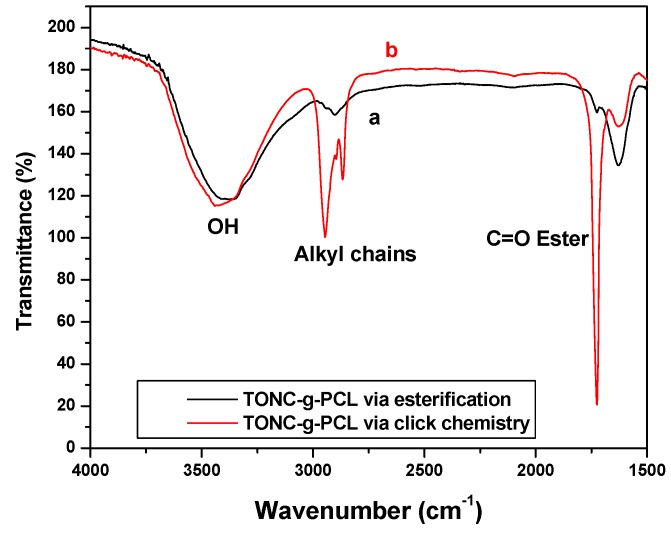
FTIR spectra of PCL (**a**) and azido-polycaprolactone (PCL-N3) (**b**).

Figure 4 shows that both methods were successful in attaching PCL to TONC surfaces. In the case of direct esterification, spectrum a, shows a very weak peak at 1725 cm^−^^1^ and no significant increase in the intensity of the peak at 2900 cm^−^^1^ which confirms the low grafting found by gravimetry (5%). This low grafting can be attributed to the large molecular weight of PCL which induces an important steric hindrance and thus affecting negatively the grafting yield. In the case of click reaction, spectrum b shows the appearance of an intense band at 1725 cm^−^^1^ typical of ester groups of PCL. Moreover, the significant increase in the intensity of the peak at 2900 cm^−^^1^ shows clearly that the steric hindrance is the main reason affecting the grafting in the case of direct esterification.

### 2.2. X-Ray Photoelectron Spectroscopy (XPS) Results

**Figure 5:**
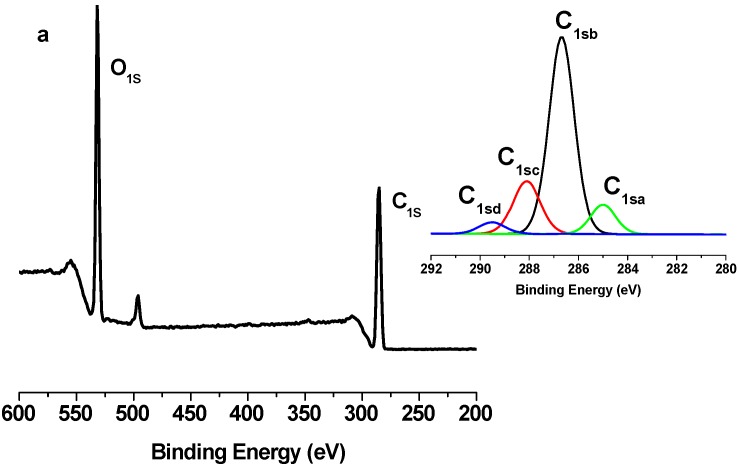
X-ray photoelectron spectroscopy (XPS) spectrum of TONC and deconvolution of its C_1s_ peak.

**Figure 6:**
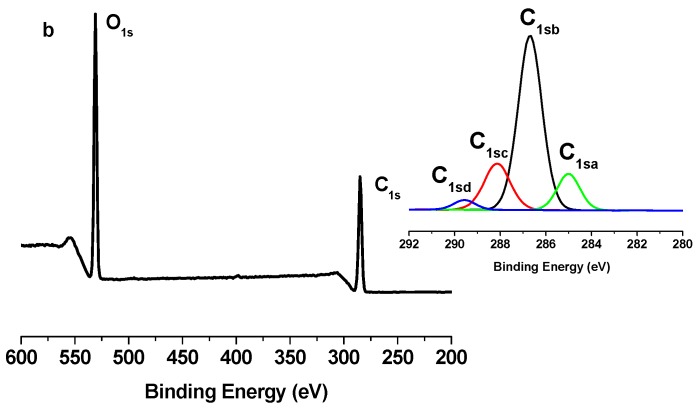
X-ray photoelectron spectroscopy (XPS) spectrum of TONC-g-PCL via esterification method and deconvolution of its C_1s_ peak.

**Figure 7:**
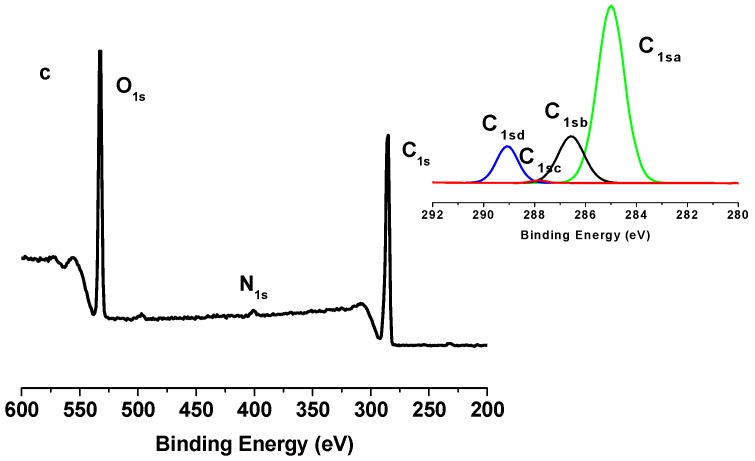
X-ray photoelectron spectroscopy (XPS) spectrum of TONC-g-PCL via click-Chemistry method and deconvolution of its C_1s_ peak.

The grafting of PCL polymer was also confirmed by XPS measurements. Figure 5, Figure 6 and Figure 7 show the XPS spectra of TONC, (TONC-g-PCL)_ester_ and (ONC-g-PCL)_click_, respectively. The carbon composition and the experimental atomic composition as determined from the XPS spectra analysis and the calculated oxygen to carbon (O/C) ratio for all samples are summarized in Table 1 and Table 2, respectively. All XPS spectra reveal that C and O are the predominant species and they occur at 285 and 532 eV, respectively. After grafting TONC with PCL by esterification (Figure 6), we did not notice a significant change in its XPS spectrum compared to that of TONC (Figure 5). This is due to the low grafting of polycaprolactone onto TONC. Whereas, in the case of grafted TONC with PCL by click-chemistry, the spectrum shows a new peak at 400 eV corresponding to the N atom (Figure 7). Additionally, in the high-resolution carbon spectra, the intensity of the C1sa peak increased from 9.86% for TONC to 70.11% for (TONC-g-PCL)_click_ (Table 1), which confirms the presence of the alkyl chain of the polymer. Likewise, the intensity of the C1sd peak increased from 4.07% for TONC to 11.46% for (TONC-g-PCL)_click_, which confirms the high quantity of grafted chains of the polyester on the TONC [33,34,35,36]. Finally, the analysis of data presented in Table 2 shows that the O/C atomic ratio of ONC decreased (from 0.49 to 0.26) as a result of PCL grafting on TONC.

**Table 1:** C_1s_ narrow scan X-ray photoelectron spectroscopy (XPS) spectra for TONC, (TONC-g-PCL)_ester_ and (TONC-g-PCL)_click_.

Binding type	C_1sa_ (C–C/C–H)	C_1sb_ (C–O)	C_1sc_ (C=O)	C_1sd _(O–C=O)
Energy (eV)	285	286.5–7	288	289.2–9
TONC	9.86	66.91	19.16	4.07
(TONC-g-PCL)_ester_	12.89	64.96	18.86	3.29
(ONC-g-PCL)_click_	70.11	17.89	0.54	11.46

**Table 2:** Experimental atomic composition and oxygen to carbon (O/C) ratio obtained by XPS analysis for TONC, (TONC-g-PCL)_ester_ and (TONC-g-PCL)_click_.

Sample	Atomic content %			O/C
	C	O	N	
TONC	67.11	32.89	0	0.49
(TONC-g-PCL)_ester_	68.46	31.55	0	0.46
(ONC-g-PCL)_click_	75.26	20.80	3.94	0.26

### 2.3. Transmission Electron Microscopy (TEM) Results

**Figure 8:**
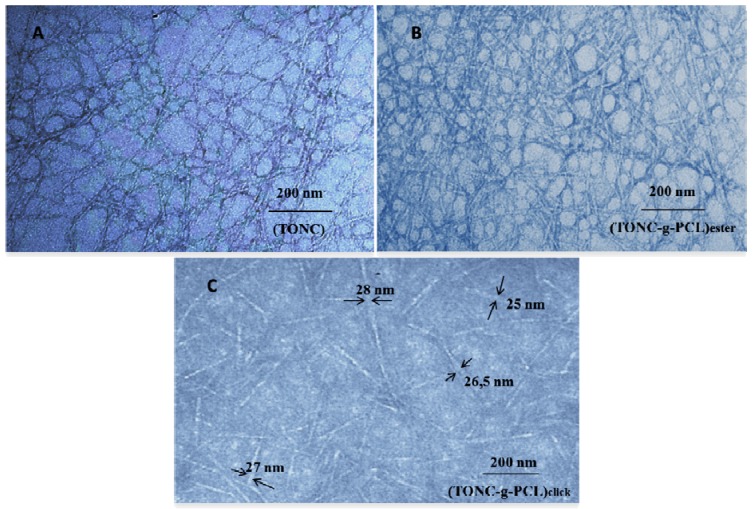
Transmission electron micrographs of: (**A**) TONC; (**B**) (TONC-g-PCL)_ester_ and (**C**) (TONC-g-PCL)_click_.

The transmission electron micrographs of TONC, (TONC-g-PCL)_ester_ and (TONC-g-PCL)_click_ are displayed in Figure 8.

Figure 8A shows that TONC fibers are individualized with a width of about 3 nm and length exceeding 1 µm. Shape and size of individualized TONC are similar to those reported by other researchers [31,40]. After grafting TONC with PCL by esterification (Figure 8B), we did not notice a significant change in the morphology of TONC, this is due to the low grafting of PCL on fibers, which confirms the ineffectiveness of esterification as a method of grafting. In the case of grafting TONC with PCL by click chemistry, the TEM image (Figure 8C) reveals a significant increase in the width of grafted TONC fibers (28 nm). The increase of the width of the grafted TONC fibrils confirms clearly that the PCL chains are incorporated onto the surface of the TONC network. In addition, the fibers became slightly agglomerated due to the decrease in polarity of TONC, which reduces the dispersion of the fibers in the polar solvents.

### 2.4. Thermogravimetry Analysis (TGA) Results

**Figure 9:**
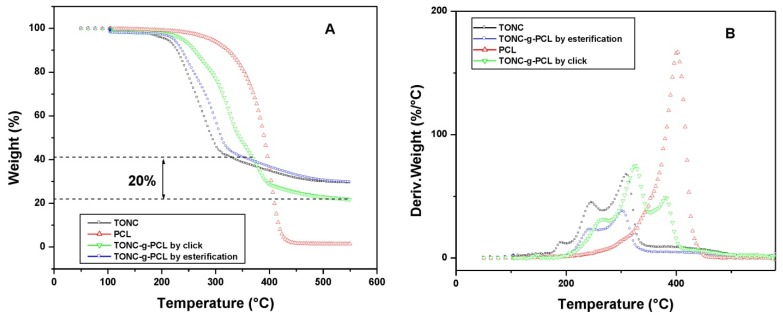
Thermogravimetry analysis (TGA) (**A**) and differential thermogravimetry (**B**) curves of PCL (red curve), TONC (black curve), TONC-g-PCL via esterification reaction (blue curve) and TONC-g-PCL via click reaction (green curve).

Additional evidence of PCL binding was obtained from the decomposition behavior in thermogravimetry as shown in the thermograms of TONC, TONC-g-PCL and PCL (Figure 9). In general, thermal decomposition of unmodified cellulose proceeds at 320–360 °C. TEMPO-mediated oxidation significantly decreases the thermal stability of native cellulose [37,38]. The present result for TONC (see black curve) was related to decarbonation of the formed anhydroglucuronic acid groups [39]. The main decomposition event of TONC is resolved into two peaks, which likely represent the decomposition of the TONC surface and core fractions. After grafting PCL onto TONC, (Figure 9 green curve), these peaks were shifted to slightly higher temperatures and a third peak appears at 370 °C characteristic of the decomposition of grafted PCL. The decrease in PCL weight ~20% in the blue curve was determined by extrapolation of the PCL component (dotted line). By assuming that the grafted PCL decomposes in the same way as in the red curve, the weight fraction of PCL in the TONC-g-PCLclick was 20%. Compared to TONC, the thermogram of TONC-g-PCL ester showed the same degradation behavior (orange curve), which confirms the low grafting of PCL by direct esterification.

### 2.5. Contact Angle Results

**Figure 10:**
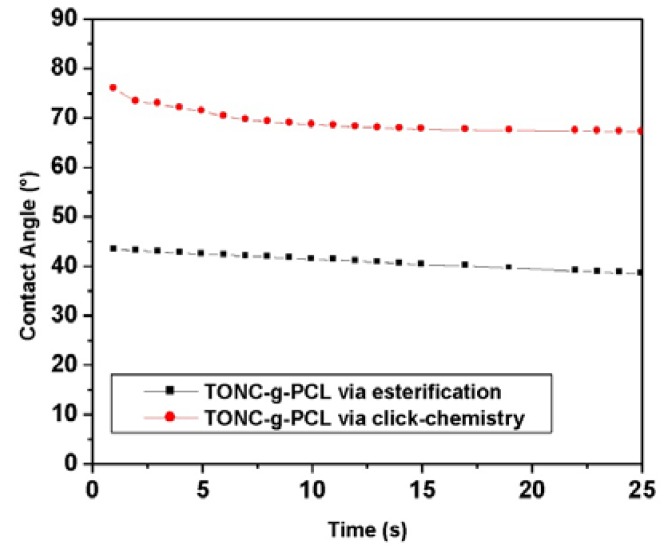
Contact angle *vs.* time performed with water for grafted TOCN samples.

**Figure 11:**
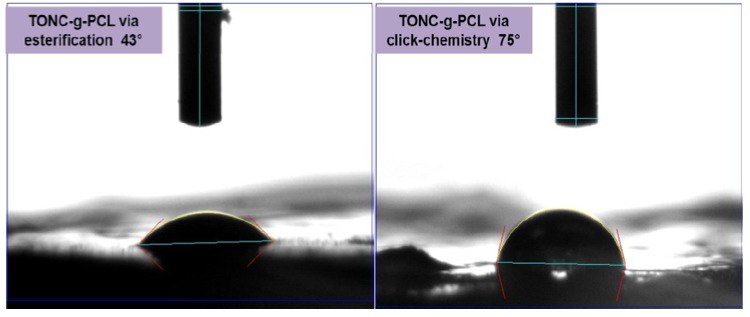
Profiles of water contact angles on TONC-g-PCL via esterification and TONC-g-PCL via click-chemistry 25 s after drop deposition.

To verify the polarity of TONC-g-PCL, we further characterized our samples using contact angle (CA) measurements which allow the estimation of the change in the hydrophobicity of the TONC-g-PCL compared to unmodified TONC. While the water droplet was rapidly adsorbed on the surface of (TONC-g-PCL)_ester_, the initial shape of the water droplet remained unchanged for a much longer period of time in the case of the (TONC-g-PCL)_click_ (Figure 10). In addition, a significant increase in contact angle values was observed for (TONC-g-PCL)click (CA~75°) compared to (TONC-g-PCL)_ester_ (CA~43°) (Figure 11), which show clearly the more hydrophobic nature of the grafted substrate produced by click chemistry compared to that produced by esterification. The contact angle values obtained for (TONC-g-PCL)_click_ is in agreement with those found in the literature [40,41]. The highest contact angle value observed for (TONC-g-PCL)_click_ is ascribed to the highest grafting efficiency, thanks to the intercalation of a spacer molecule, that moves away the reactive functions from the surface of TONC and, consequently, makes them more accessible for further grafting with high molecular weight grafts.

## 3. Experimental Section

### 3.1. Materials

A Commercial never-dried bleached kraft pulp was used as the starting material for the oxidized nanocelluloses, 10-Undecyn-1-ol (96%), magnesium sulfate (MgSO_4_), sodium bicarbonate (NaHCO_3_), polycaprolactone-diol (Mn 2000; Sigma-Aldrich), p-toluenesulfonyl chloride (TsCl), sodium azide (NaN_3_), sodium L-ascorbate, copper (II) sulphate pentahydrate (CuSO_4_(H_2_O)_5_) and NaBr were purchased from Sigma-Aldrich. Sodium hypochlorite (NaOCl) was procured locally. Chemicals and solvents were commercial products used as received.

### 3.2. Preparation of Oxidized Nanocelluloses TONC by Ultrasound-TEMPO-NaBr-NaOCl-Oxidation

According to the method of Mishra *et al.* [42], the oxidation was carried out in a specially designed glass reactor placed in an ultrasonic bath. The glass reactor held 20 g (wt.%) pulp sample (1% pulp consistency) in deionized water. The reaction was carried out under a frequency equal to 170 kHz, at 1000 W of ultrasonic power intensity. Both 4-acetamido TEMPO (0.46 g, 0.11 mmol per g cellulose fiber), and NaBr (1.27 g, 0.617 mmol per g cellulose fiber) were dissolved in 50 mL of de-ionized water and added to the fiber suspension. A pH-stat was used to maintain the pH using 0.5 M NaOH or 0.5 M HCl. The oxidation process was started by adding the desired amount of the NaOCl solution (3.75 mmol NaOCl per g of cellulose fiber), at 25 °C. The reaction was stopped after 90 minutes by adding 50 mL of ethanol, and the final pH of the solution was adjusted to 7.0 by adding 0.5 M NaOH or 0.5 M HCl as required. The US-TEMPO-oxidized cellulose slurry was filtered, thoroughly washed with de-ionized water, and preserved at 4 °C.

### 3.3. Measurement of Carboxyl Group Content

The carboxylic content in the oxidized cellulose was determined using the conductometric titration method using a Dosimat 765 (Metrohm) titrator according to the technique of Saito and Katz [41,43]. In this procedure, the sodium carboxylate groups in the TEMPO oxidized celluloses were converted to the free carboxyl form by treating the sample with 0.1 M HCl solution three times and, finally, thoroughly washing with de-ionized water to remove the excess acid. The oxidized pulp prepared in this way was transferred to a 600 mL beaker containing 450 mL of 0.001 N NaCl solution and mixed well. Five millilitres of 0.1 N HCl was added to the fiber suspension before starting titration of the carboxylate groups with 0.1 N NaOH solution. At the end of the titration, the fibers were filtered under vacuum, washed and dried in an oven at 105 °C to determine the exact weight of the sample. The carboxyl content expressed in mmol/g was calculated by the software (Soft Imaging System).

### 3.4. Production of TEMPO-Oxidized Nanocelluloses (TONC)

The oxidized cellulose sample (0.4 g) was suspended in water (400 mL) at 0.1% concentration. The slurry was mechanically homogenized by means of blender for a total time of 20 min [44]. The obtained suspension was centrifuged at 10,000× *g* for 15 min in order to separate oxidized nanocelluloses (supernatant) from the non-fibrillated fraction.

### 3.5. Synthesis of Click Precursor Bearing Alkyne Groups (TONC-Undecynoate)

The grafting of 10-Undecyn-1-ol onto TONC was achieved in two steps. In the first step, 4.1 g of 10-Undecyn-1-ol (4.67 mL) were added to triethylamine (2.30 g) and 4-dimethylaminopyridine (0.40 g). The mixture was dissolved in CH_2_Cl_2_ (50 mL), and the solution was cooled over an ice bath. Tosyl chloride (8.00 g, 42 mmol) dissolved in CH_2_Cl_2_ (50 mL) was slowly added to the mixture over a period of 40 min. After 4 h at room temperature, the mixture was poured onto saturated aq. NaHCO_3_ (200 mL). The crude product was extracted with CH_2_Cl_2_, dried over anhydrous MgSO_4_, filtered and concentrated under vacuum. The prepared product (11-(4-Methylbenzenesulfonyl)-1-undecyne) was then weighed and a yield of 30% was determined. In the second step, 2 g of 11-(4-Methylbenzenesulfonyl)-1-undecyne) was added to 1000 mL of TONC (0.2% *w*/*v*) under moderate stirring at 50 °C. After 36 h, chemically modified TONC-Undecynoate was isolated by filtration and poured into 1L of water. Then purification was performed by successive washing with water and ethanol and drying at 50 °C, for 48 h before being characterized.

### 3.6. Synthesis of p-toluenesulfonylpolycaprolactone (PCL-OTs)

As proposed by Krouit *et al.* [34,45], 11.44 g of TsCl (60 mmol, 5 equiv/PCL-diol) was dissolved in 40 mL of THF and added dropwise to a stirred solution containing PCL (24 g, 12 mmol), Et3N (41.5 mL, 0.3 mol, 25 equiv/PCL-diol), trimethylamine hydrochloride (573 mg, 6 mmol, 0.1 equiv/TsCl) in 40 mL of tetrahydrofuran (THF) at room temperature. The mixture was stirred for 1 day. Insoluble products were filtered out, and the clear reaction mixture was poured into a bath of ethyl ether at 0 °C. The precipitated product, p-toluenesulfonyl-polycaprolactone (PCL-OTs), was recovered and dried under vacuum. The prepared polymer was then weighed, and a yield of 70% was determined.

### 3.7. Synthesis of Azido-polycaprolactone (PCL-N3)

One gram of sodium azide was added to a solution of PCL-OTs (15 g) in 40 mL of dimethylformamide (DMF) under moderate stirring at room temperature for 24 h. The mixture was then filtered to remove insoluble products, and the ensuing filtrate was poured into hexane at 0 °C. The precipitated azido-polycaprolactone (PCL-N3) was recovered and dried under vacuum. The reaction yield was about 80%.

### 3.8. Esterification of TONC by PCL-OTs [(TONC-g-PCL)_Esterification_]

**Figure 12:**
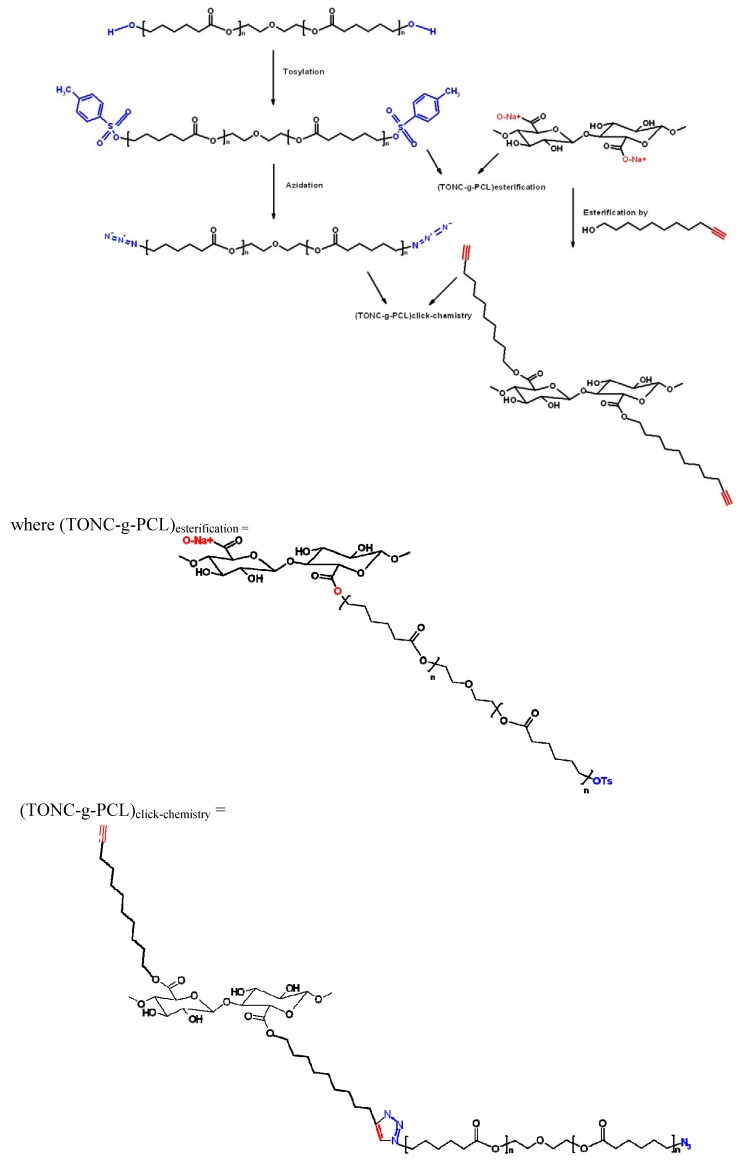
Strategies adopted for the grafting of polycaprolactone-diol onto TONC by direct esterification and click chemistry.

1.62 g of *p*-toluenesulfonylpolycaprolactone was dissolved in 15 mL of ethanol and added to a stirred aqueous suspension of TONC (500 mL 0.2%) at room temperature. After 36 h reaction, the mixture was filtered to remove sodium tosylate and unreacted PCL-OTs and the purification was then performed by successive washing with water and methanol. Finally, the grafted product was recovered and dried under vacuum before being characterized. The adopted strategy is presented in Figure 12.

### 3.9. Grafting of PCL-N3 onto TONC-Undecynoate by Click Chemistry [(TONC-g-PCL)_click-chemistry_]

One gram of cellulose undecynoate fibers was added to a solution of azido-polycaprolactone (1.620 g, 1.23 mmol) in 40 mL of THF, to which freshly prepared solutions of sodium ascorbate (250 µL, 0.25 mmol, 1 M) in water and a 75% solution of copper (II) sulfate pentahydrate in water (170 µL, 0.05 mmol) were added. The heterogeneous mixture was stirred in the absence of light, at room temperature. After 36 h reaction, the grafted cellulose fibers were filtered and washed with CH_2_Cl_2_ and water. After successive Soxhlet extraction with methylene chloride and water, the fibers were recovered, dried at 50 °C for 48 h before being characterised. The adopted strategy is presented in Figure 12.

## 4. Characterization

### 4.1. Fourier Transform Infrared Spectrometry (FTIR)

Two-percent *w*/*w* of dried sample was mixed with KBr, and pellets of the mixture were made. FTIR spectra were recorded using a Perkin-Elmer System 2000 in transmission mode. A total of 32 scans were taken per sample with a resolution of 4 cm^−1^ (4000–400 cm^−1^).

### 4.2. X-Ray Photoelectron Spectroscopy (XPS)

XPS experiment was carried out using a Kratos Axis Ultra spectrometer equipped with a monochromatic Al Kα X-ray source (*E* = 1486.6 eV) with a power of 225 W. Samples were placed in an ultrahigh vacuum chamber (10^−9^ torr at room temperature) with electron collection by a hemispherical analyzer at a 90° angle. The overall spectrum was shifted to ensure that the C–C/C–H contribution to the C1s signal occurred at 285.0 eV. Gaussian peak profiles were used for spectral deconvolution of C1s spectra.

### 4.3. Transmission Electron Microscopy (TEM)

Drops of the suspensions were deposited onto glow-discharged carbon-coated electron microscopy grids. The excess liquid was absorbed by a piece of filter paper, and a drop of 2% uranyl acetate negative stain was added before drying. The liquid in excess was wiped off, and the remaining film of stain was allowed to dry. The specimens were observed using a Philips EM 208S microscope operating at 80 kV. The size of the fibers was measured from digital images using the Soft Imaging System SIS.

### 4.4. Thermogravimetry Analysis (TGA)

Thermal stability analysis (TGA and DTGA) of the samples was carried out in a Perkin-Elmer (Pyris Diamond) Thermoanalyzer. Samples of pure ONC, PCL, and composites were heated in open platinum pans from 50 to 575 °C, under a nitrogen atmosphere, at a heating rate of 5 °C/min. Then samples were heated from 575 to 950 °C under air at a heating rate of 15 °C/min.

### 4.5. Contact Angle (CA)

CA measurements were carried out on pellet sample before and after treatment in order to determine the change in wettability. The water sessile drop contact angle (CA) measurements were carried out on our substrates using an FTA4000 Microdrop Instrument (First Ten Angstrons, Portsmouth, VA, USA) equipped with a CCD camera. All measurements were performed eight times for each sample.

## 5. Conclusions

Successful surface grafting of polycaprolactone onto tempo-oxidized nanocellulose fibers has been achieved by two methods: esterification and click-chemistry. Compared to esterification treatment, click chemistry leads to enhanced grafting of nanocellulose, thanks to the intercalation of a spacer molecule, which moves away the reactive functions from the nanofiber’s surface and makes them more accessible for further grafting with polycaprolactone. Consequently, the obtained PCL-grafted nanocellulose by click reaction, compared to that obtained by esterification, demonstrated significant improvements in term of surface polarity when dispersed in non-polar solvents. Work is in progress in order to investigate both treatments at different reaction conditions, and to study the mechanical properties of the obtained products. The hydrophobic properties of the grafted TOCN material could be suitable to be used as reinforcement for nonpolar polymer matrices for several applications.
